# Supplementary material for: Sickle cell disease in Cameroon: Taking out the “neglect” and highlighting key opportunities for sustainable control
Source: PLOS Glob Public Health. 2024 Oct 24;4(10):e0003668. doi: 10.1371/journal.pgph.0003668 (PMC11501032; doi:10.1371/journal.pgph.0003668)
Supplement: S1 Table — (DOCX) [file pgph.0003668.s001.docx]

SI Table 1: Description of included documents in the documentary analysis.

| **#** | **Title** | **Author/Year** | **Document type** | **Description** |
| --- | --- | --- | --- | --- |
| 1 | Sickle Cell Disease in Cameroon. Care guide. MINSANTE; 2021. | Ministry of Public Health - Cameroon (2021) | National Health Policy document | This document established a clear diagnosis and management of SCD in Cameroon. |
| 2 | Progress in The Implementation of The African Region Sickle-Cell Strategy 2010–2020. Africa: 2020. | World Health Organization (2020) | International evaluation report | This report presents an evaluation of WHO’s proposed policies on SCD for its AFRO region countries, of which Cameroon is part |
| 3 | Sickle-Cell Disease: A Strategy for the WHO African Region. | World Health Organization (2010) | International Health Policy document | WHO SCD strategy for the Africa Region partner countries |
| 4 | WHO, Africa Health Observatory. Health Analytical Profile 2016, Cameroon. Yaounde, Cameroon: | MINSANTE (2016) p. 143 (page 83). | National Health Policy document | National health analytical profile |
| 5 | District Health Information Software (DHIS2) | Ministry of Public Health- Cameroon (Accessed, 2024) | National Health information systems report | The DHIS2 presents key health routine data regarding coverage and access to health services over the national territory which is used to guide programs and intervention plans |
| 6 | CBC Health Services brings Sickle Cell Disease to Limelight [Internet]. CBC Health Services brings Sickle Cell Disease to Limelight. | Cameroon Baptist Convention, 2021 | Blog + News report | This blog and news flash reports on the International SCD day activities carried out by the CBC health center in Yaounde around sensitization and free screening |
| 7 | Defining global strategies to improve outcomes in sickle cell disease: a Lancet Haematology Commission. | Piel F, Rees D, DeBaun M, Nnodu O, Ranque B, Thompson A. (2023) | Journal article | This article reports on a recent assessment of the global state and care offer for SCD. Offering key recommendations and strategies for moving forward. Most of which are a base for key policies which could be adapted across contexts and in Cameroon. |
| 8 | Global, regional, and national prevalence and mortality burden of sickle cell disease, 2000–2021: a systematic analysis from the Global Burden of Disease Study | Thomson AM, McHugh TA, Oron AP, Teply C, Lonberg N, Vilchis Tella V, et al. (2023) | Journal article | This article reports on the global modelled epidemiological estimates for SCD. This fills a gap on epidemiological data especially in sub-Saharan Africa. |
| 9 | Malaria Indicator Survey (page 63-64). | National Institute of Statistics (NIH), National Malaria Control Program (NMCP), The DHS Program, ICF (2023) | National survey report | The report presents the most recent survey on malaria and its indices. This is relevant to our study as malaria is a key indicator for SCD prevalence and management in the region |
| 10 | Cameroon SCD landscape | CHAI Cameroon (2024) | SCD Landscape report | Preliminary research report from SCD landscape |
