# Supplementary material for: Sickle cell disease in Cameroon: Taking out the “neglect” and highlighting key opportunities for sustainable control
Source: PLOS Glob Public Health. 2024 Oct 24;4(10):e0003668. doi: 10.1371/journal.pgph.0003668 (PMC11501032; doi:10.1371/journal.pgph.0003668)
Supplement: Attachment — Submitted filename: 240707-Response to Reviewers.docx [file pgph.0003668.s002.docx]

**Rebuttal Letter**

**Manuscript Reference**: PGPH-D-24-00845
**Manuscript Title**: Sickle Cell Disease in Cameroon: Taking out the “neglect” and highlighting key opportunities for sustainable control

**Dear Editor,**

Thank you for your consideration of our manuscript, and for the key changes proposed here to improve its readability and overall impact.

**Reviewer #1**

Thank you for your detailed review of our manuscript and for the key inputs. We have now considered you raised points, and detail our changes/responses here:

****The description of the study design and data collection methods lack sufficient detail.**

- Thank you for highlighting this. We have now added more details on study design and data collection methods, as seen in lines 87-91, to further complement and highlight that which we had earlier presented. This clarifies better the qualitative approach and the precise tools used within this approach and how they are used.

****There are inconsistencies in terminology, such as switching between “SCD” and “SCT” without clear definitions or distinctions.**

- We have now reviewed this within this version of our manuscript, and early enough in the introduction, defined better the term “Sickle cell trait”, as seen on lines 54-56, and 67-68. This definition given here differentiates “SCT” from “SCD” which we had defined on lines 42-43, and again have added some specification on lines 67-68. Thank you for highlighting this aspect.

****The recommendations are broad and lack specific implementation strategies**

- Thank you, we have now added details to our recommendations, especially around the level of implementation within the Cameroonian health system levels. In addition to this, we highlighted some specifications on existing programs and activities within primary health care which could be considered. These are detailed on lines 451-583 (Table 2)

**Reviewer #2**

Thank you very much for your time and this review which you have accorded our manuscript. We have considered your feedback and have detailed our response as follows:

**** Good study that could assess the current scenario of SCD and highlight the need for policy level actions. If approximately 7000 children are born annually in the country, more quantitative data analysis would definite be helpful.**

- Thank you very much for bringing up this point. We acknowledge the importance and added value an analysis of quantitative data would have given our manuscript. But please, at this time (for reasons related to the costs of carrying out a large-scale quantitative study, and our preferred methodology), we have limited our contribution to this structure and the qualitative data presented within this manuscript. This qualitative data reflects the individual perceptions of policy makers, health workers and teenagers (and their parents) living with the sickle cell disease and offers an in-depth narrative and angle which we think identifies key gaps and could offer a start point for reflection and action, which further studies can leverage for carrying out large scale quantitative studies. But we have considered this lack of quantitative data as limit within our study and have emphasized this within this new version of our manuscript as you will find on lines 585-587. Thank you again for highlighting this.
